# The Mystery of a Lost Limb

**Published:** 1891-07

**Authors:** Herman Fascher


### THE MYSTERY OF A LOST LIMB.

By Herman Fascher.

A recent article in *The Progressive Thinker*, headed "A Deep Mystery," caused me to ask a neighbor of mine, Mr. Seth A. Pymm whose right arm was amputated during his youth just below the elbow, about his opinion and experience.

When I read the above article to him, he at once said: That's true, every word of it. I have had the same sensation myself, and have it now. My arm was crushed in a cane mill, and after being amputated, was given to a young man for burial, a brother-in-law keeping the thumb nail which was torn out. The young man wrapped the arm, with the hand attached, in a cloth, and buried it some six miles south of Salt Lake City, near a peach tree, as he said.

While the young man was gone, I could feel the hand of the amputated arm hanging down and receiving a shock, which was occasioned (as was ascertained afterwards) by the young man taking the arm under his arm and jumping out of a buggy. Soon after, I could feel the arm and hand being put in a cramped position, which caused me considerable uneasiness, and immediately after I could feel the dirt being trampled upon it, which caused me to cry out with pain. The young man did not return, but went off some where else, and when afterwards asked to locate the spot, could not find it again, as there were a great many peach trees in the neighborhood. Well, I suffered terribly on account of this careless interment, and although I had a small coffin made, and searched the country for the missing member, it was never found, and the coffin remained empty.

Well, to this day, after the lapse of nearly thirty years (it happened in 1862), I always feel my lost hand in that cramped position; not only that, but sometimes it feels cold, and it seems that it is impossible for me in cold weather to put clothing enough on my stump to keep the lost hand warm. This feeling of coldness can only be relieved by rubbing the stump above the elbow, and stroking it downward toward the missing member, as holding it near a fire has no effect whatever. Sometimes the hand itches; at other times it seems to be asleep, as the popular expression is. Both of these sensations can only be relieved by rubbing the stump.

Another thing: When I was in England in 1865, three years after the amputation, I was walking along the street with a friend, when all at once I screamed out loud with pain in my missing thumb, and I said to my companion: "Confound it, that fool of a brother-in-law of mine is scraping my thumb nail." I put down the date and hour when this occurred, and when I returned to Salt Lake City, I at once inquired of my brother-in-law what caused him to scrape my thumb nail.

He was quite astonished at my question, but admitted frankly that about the time I felt the pain, he happened to look at the nail, and seeing a small piece of the "quick" of the flesh still adhering, took his pocket knife and scraped it off; and that was exactly the nature of the pain I felt, and it was as intense as if it were being done on a healthy thumb. I also know of a relative of mine whose leg was amputated above the knee, and who some years afterward felt an itching sensation in his missing foot, as he was sitting on the edge of the bed, and forgeting all about it being amputated, reached down to relieve himself by scratching, when, loosing his balance on account of the missing member, fell forward and broke his shoulder.

I consider this positive evidence of the existence of a spiritual body co-extensive and co-existing with the physical body. There is no question about the physical amputated arm of Mr. Pymm being decomposed into its constituent elements long ago, hence any sensations of cold or itching, or being "asleep," cannot originate in the physical arm and be communicated by "sympathy," as was the pain of the scraping of the thumb nail and the trampling of dirt upon the arm during burial.

It is evident that all sensations must originate with the ever present arm of the spiritual body. Mr. Pymm declares that he can now move every muscle and every finger of the missing hand the same as if it were there, and, in fact, he says "It is there." Although he cannot feel physical bodies with it, if he attempts to grasp it, it seems to slip away from him and draw itself up into the stump; but when he touches and handles the stump it returns to its normal position. What other explanation can be found for these phenomena except that man is at least a dual personality, having a physical and metaphysical, or spiritual body, both at the same time? But who will assert that this spiritual body is the ultimate, the innermost individuality of man?

Is it not more reasonable to suppose that the spiritual body serves the same purpose on the spiritual plane that the physical body does on the physical plane? being merely a means to an end, which is the collection of experiences for the education and endless progression of the innermost self, the divine over soul? Do not spirits tell us that on progressing from one general sphere to another, they go through a similar experience as they did when passing from the physical to the spiritual plane? That portions of their spiritual bodies, which are too crude to enter the higher sphere, are thrown off during a state of unconsciousness, and these spirits are thereby rendered invisible to the friends who remain behind ? Does this not prove that man is a vastly more complicated being than is generally supposed, even by Spiritualists? Does this not prove that the innermost individuality is superior to, and independent of, any and all of these bodies? If so, then even the most refined and attenuated of the spiritual bodies must finally pass away, and must be thrown off, as too cumbersome and too imperfect to further serve the soul, which thereafter may, for the first time perfectly and indissolubly unite with its equally perfect soul-mate ; two half souls, two half spheres joined together in perfect harmony form one truly perfect sphere, one truly perfect soul, a centre of almost infinite power, love and wisdom, a God.

